# Mining Tamarix ramosissima roots for endophytic growth promoting fungi to improve wheat root growth

**DOI:** 10.21203/rs.3.rs-4277791/v1

**Published:** 2024-04-29

**Authors:** Mostafa Ebadi, Solmaz Najari, Leila Zarandi Miandoab, Nader Chaparzadeh, Ali Ebadi

**Affiliations:** Azarbaijan Shahid Madani University; Azarbaijan Shahid Madani University; Azarbaijan Shahid Madani University; Azarbaijan Shahid Madani University; Nuclear Science and Technology Research Institute

**Keywords:** Ascomycota, Aspergillus fumigatus, Endosphere, IAA, Root developing

## Abstract

Endophytic fungi are commonly found in the root endosphere and can enhance plant growth through various mechanisms. The aim of this study was to isolate cultivable endophytic fungi associated with the roots of *Tamarix ramosissima* and to evaluate their plant growth promoting properties. About 35 isolated fungal endophytes belonging to the Ascomycota from four different genera were isolated from the endosphere of *T. ramosissima: Alternaria, Aspergillus, Fusarium* and *Talaromyces*. These fungal endophytes showed different abilities to solubilize phosphate and produce indole-3-acetic acid (IAA). The fungal isolates of *T. allahabadensis* (T3) and *A. niger* (T4) showed different efficiency in solubilizing phosphate. Almost all fungal isolates were able to produce IAA, and the highest value (0.699 μg/ml) was found in the isolate of *F. solani* (T11). Inoculation of wheat seeds with endophytic fungi significantly increased the initial growth of wheat roots. The results showed that inoculation with the endophytic fungus *A. fumigatus* T15 significantly increased root length by 75%. The extensive root system of *T. ramosissima* may be due to symbiosis with IAA-producing endophytic fungi, which enhance root development and water uptake in dry conditions. These fungi can also boost soil phosphorus levels, promoting plant growth.

## Introduction

Phosphorus is one of the most important limiting factors for plant growth and one of the most immobile and least available nutrients. Its deficiency leads to a drastic decline in plant productivity in most soils. Phosphorus is one of the components of DNA, RNA and energy-carrying molecules in plants and plays an essential role in biochemical processes^1^. Phosphorus is a macronutrient and farmers use large amounts of chemical phosphorus fertilizers to achieve optimal crop production. Due to the low mobility of phosphorus in the soil, the efficiency of the chemical fertilizers used is very low (10–25%) and their frequent use to compensate for this deficiency not only leads to a decrease in economic productivity, but also to environmental pollution^2^. A reduction in the efficiency of phosphate fertilizers in agricultural soils is caused by the high reactivity of phosphorus with some soil elements such as calcium and iron and the formation of insoluble phosphorus minerals^3^. Elias et al. reported that 95–99% of soil phosphorus is in the form of insoluble minerals that are not available to plants^4^. An alternative approach to reduce the consumption and increase the efficiency of chemical fertilizers is to use indigenous microflora, especially those that can dissolve insoluble mineral elements in the soil. Some soil microorganisms can dissolve insoluble phosphorus minerals and increase their availability in the soil^5^. In the past three decades, phosphate-dissolving microorganisms have been reported and the phosphate-dissolving potential of various bacteria and fungi has been evaluated. Among the phosphate-dissolving microorganisms, *Pseudomonas* and *Bacillus* species are the most common among bacteria and *Aspergillus* and *Penicillium* species are the most common among fungi. Among these microorganisms, the ability of phosphate-dissolving fungi is greater than that of bacteria, as they have a hyphae-like structure and spread deeper in the soil^6^. These microorganisms also have other properties, such as the ability to produce phytohormones that can improve plant growth^7^.

Phytohormones are organic compounds that are involved in plant growth, physiological processes and defense mechanisms. The microbiome associated with plants can alter phytohormone homeostasis and manipulate physiological responses under environmental stress. Indole-3-acetic acid (IAA or auxin) is one of the phytohormones that stimulate plant growth. It plays an important role in increasing root biomass and the uptake of plant nutrients^6^. The importance of stimulatory substances to improve plant growth has been investigated in recent years. Endophytic fungi can improve and increase plant growth by producing growth-stimulating substances such as auxin^8,9^. The ability of fungi to produce auxin varies, and even different isolates of the same species can differ in this respect^10^. Many physiological processes, including growth, cell differentiation, germination, vascular tissue formation, photosynthesis, root elongation, etc. are affected by auxin. IAA, which is produced by endophytic fungi, is responsible for stimulating root growth, nitrogen uptake, increasing photosynthesis and expanding host growth in the presence of many abiotic stress factors. For example, auxin produced by *Phoma* and *Penicillium* increases host growth and the uptake of essential nutrients such as calcium, potassium and magnesium under salt stress. Due to the high pH and abundance of calcium ions in many Iranian soils, the available form of phosphorus is primarily below the threshold and required level for plant growth. The use of native phosphate solubilizing strains is a biological approach to solve this problem. Studies have shown that the positive interaction of the host plant with fungal endophytes can increase the plant’s tolerance to biotic and abiotic stress factors^11^. Thanks to their functions such as dissolving phosphate, producing the hormone auxin, siderophores, increasing the synthesis of biological compounds, helping to regulate osmosis, regulating stomata, increasing the absorption of minerals and altering nitrogen accumulation, endophyte fungi improve the growth and adaptation of plants to biotic and abiotic stress conditions^12^. Therefore, it is necessary to identify and utilise these microorganisms to increase phosphorus absorption in the root zone and achieve sustainable agriculture.

*Tamarix* belongs to the Tamaricaceae family and comprises approximately 54 species, primarily distributed in countries such as Pakistan, Afghanistan, Iran, Turkmenistan, South Kazakhstan, Western China, and the Eastern Mediterranean regions^13^. *Tamarix* species exhibit high adaptability to climate change, particularly in arid and semi-arid environments. Consequently, these species host a variety of microbial endophytes that enhance plant tolerance to both biotic and abiotic stressors. Despite this, no studies have been conducted to assess the potential plant growth-promoting properties of endophytic fungi isolated from *Tamarix* species in Iran. This study aimed to isolate and identify culturable endophytic fungi from *Tamarix ramosissima* roots and characterize their growth-promoting traits.

## Results

### Morphological and Phylogenetic analysis of endophytic fungi

In this study, it was found that the roots of *T. ramosissima* were associated with endophytic fungi. A total of 35 fungal endophytes were isolated from the root fragments and classified into 9 groups based on their morphological characteristics, such as colony shape, surface texture, growth rate, and colony color (both obverse and reverse sides) (Table 1). The fungal isolates exhibited circular colony shapes, except for T4, 7, 8, and 12, which had irregular shapes on PDA. The isolates displayed abundant uniform to cottony mycelium with growth rates ranging from 2.1 to 7.3 cm after 5 days of incubation at 25°C. The colony colors on both obverse and reverse sides of the isolates varied from white to black and cream to tan on PDA.

**Figure 1:**
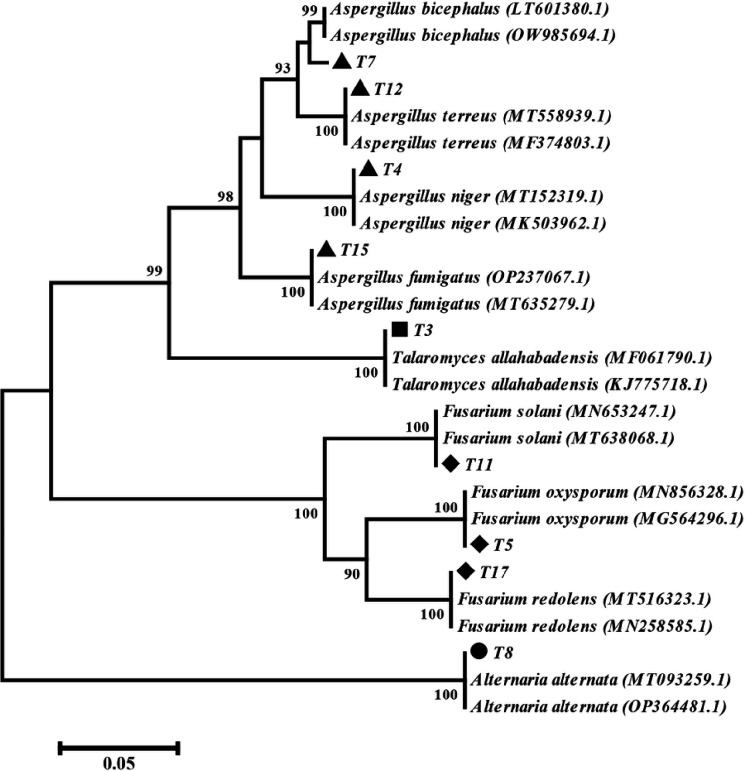
Phylogenetic analysis of endophytic fungi isolated from T. ramosissima based on the internal transcribed spacer. The phylogenetic tree was constructed using the neighbor-joining method (1000 bootstrap replications).

PCR amplification of the ITS rDNA region produced a single band ranging from 500 to 600 bp in size. The phylogenetic tree analysis revealed that the isolates could be categorized into four main clades representing different genera: Alternaria (T8), *Aspergillus* (T4, 7, 12, and 15), *Fusarium* (T5, 11, and 17), and *Talaromyces* (T3). BLAST analysis of the fungal isolates showed a 99–100% identity with ITS sequences of related species (Table 2). Therefore, based on the ITS sequence analysis, the fungal isolates were identified as follows: *A. alternata* (T8), *A. niger* (T4), *A. bicephalus* (T7), *A. terreus* (T12), *A. fumigatus* (T15), *F. oxysporum* (T5), *F. solani* (T11), *F. redolens* (T17), and *T. allahabadensis* (T3) (Fig. 1).

### Indole-3-acetic acid production ability of isolates

**Figure 2:**
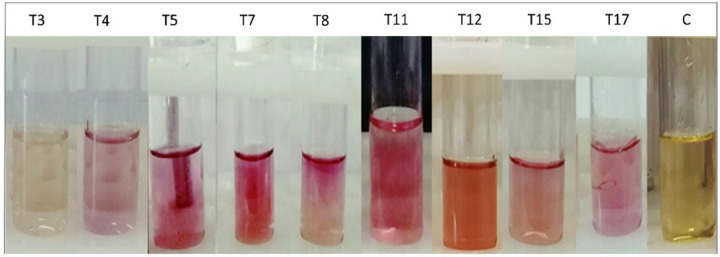
Qualitative production of IAA by fungal endophytes isolated from *T. ramosissima*. T3: *T. allahabadensis*, T4: *A. niger*, T5: *F. oxysporum*, T7: *A. bicephalus*, T8: *A. alternata*, T11: *F. solani*, T12: *A. terreus*, T15: *A. fumigatus*, T17: *F redolens*

The potency of endophytic fungal isolates for IAA production was quantitatively evaluated using the colorimetric method. When Salkowski reagent was added to the Czapek Dox medium, almost all fungal isolates (except *T. allahabadensis*) developed a pink color to varying degrees (Fig. 2). IAA production by the isolates in the Czapek Dox medium containing 5 mg/ml of L-tryptophan ranged from 0.04 to 0.699 μg/ml. The highest amount of IAA was produced by *F. solani* (T11) and *A. terreus* (T12), while the lowest value was obtained by *T. allahabadensis* (T3).

### Phosphate solubilization activity of isolates

**Figure 3:**
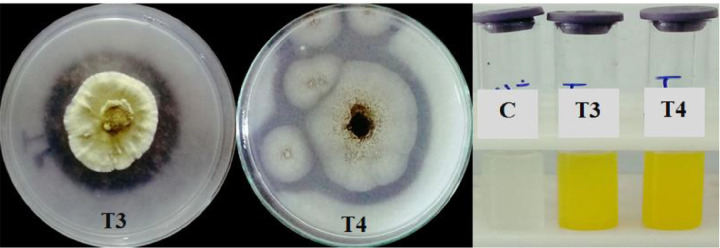
Phosphate solubilizing activity endophytic fungi obtained from *T. ramosissima* on Sperber medium. T3: *T. allahabadensis*, T4: *A. niger*

**Figure 4:**
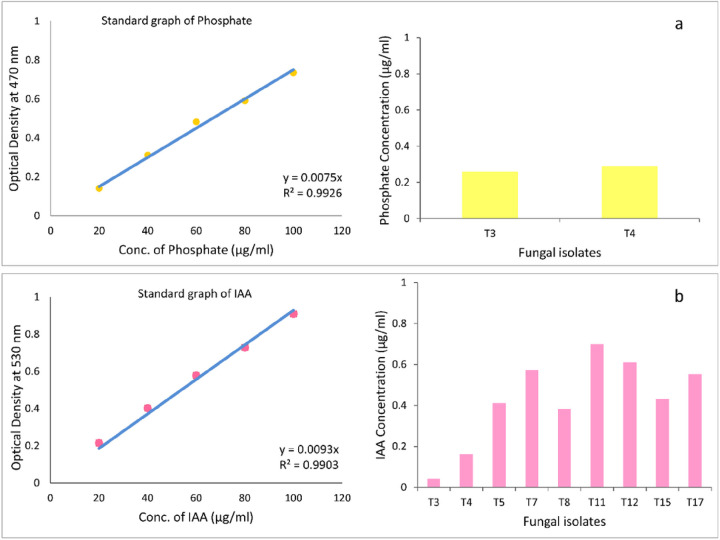
Phosphate solubilization (a) and IAA production (b) by endophytic fungi isolates. T3: *T. allahabadensis*, T4: *A. niger*, T5: *F. oxysporum*, T7: *A. bicephalus*, T8: *A. alternata*, T11: *F. solani*, T12: *A. terreus*, T15: *A. fumigatus*, and T17: *F. redolens*.

In this study, the phosphate solubilizing activity of all fungal isolates was qualitatively assessed on Sperber medium supplemented with Ca_3_(PO_4_)_2_ as an inorganic phosphate source. Among the endophytic fungal isolates, only two isolates, *T. allahabadensis* (T3) and *A. niger* (T4), exhibited clear zones, indicating their phosphate solubilizing ability. *T. allahabadensis* (T3) showed the highest solubilization zone with a diameter of 8 mm (Fig. 3). A total of 10 isolates were tested for the quantitative estimation of phosphate solubilizing activity in the Sperber medium. Upon addition of the Vanadate-molybdate reagent, only the culture medium of *T. allahabadensis* (T3) and *A. niger* (T4) changed color to bright yellow. The maximum phosphate dissolution was observed in *T. allahabadensis* (T3) and A*. niger* (T4) isolates, with concentrations of 0.260 and 0.298 μg/ml, respectively (Fig. 4).

### Effect of Endophytic Fungi Inoculation on Wheat Root Growth

**Figure 5:**
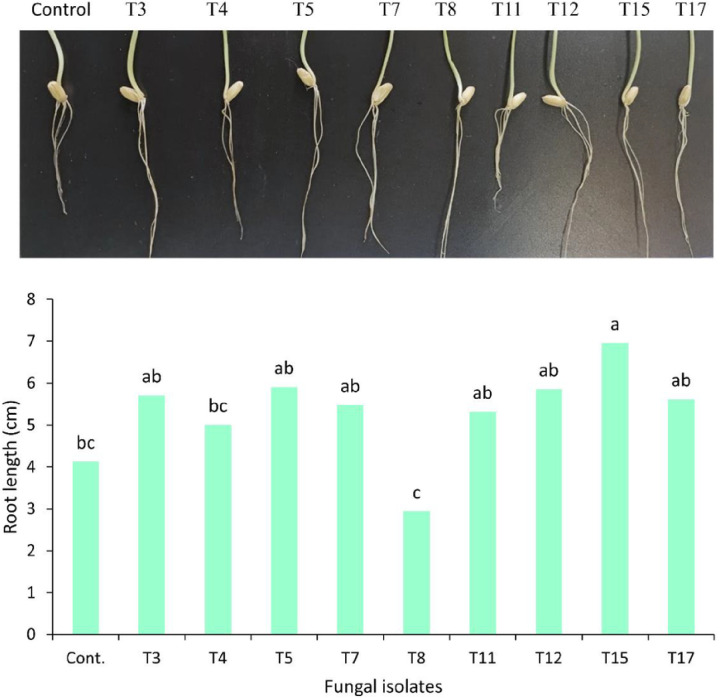
Effect of endophytic fungi as bioinoculant on root length of wheat. T3: T*. allahabadensis*, T4: *A. niger*, T5: *F. oxysporum*, T7: *A. bicephalus*, T8: *A. alternata*, T11: *F. solani*, T12: *A. terreus*, T15: *A. fumigatus*, and T17: *F. redolens*. Different letters (a, b, c, and d) on bars denote that mean values are significantly different (p < 0.05) by the Tukey LSD test.

The data analysis revealed a significant increase in wheat root length with the bioinoculation of endophytic fungal isolates (Fig. 5). Specifically, inoculation with fungal endophyte *A. fumigatus* T15 led to a significant enhancement in root length compared to the control (non-inoculated) and fungal treatments of *A. niger* T4 and *A. alternata* T8 (p < 0.05). However, other fungal isolates did not show a significant effect on root length. In fact, inoculation with fungal *A. alternata* T8 resulted in a significant reduction in root length compared to the other treatments.

## Discussion

Isolation and identification of endophytes are crucial steps in studying phylogeny, diversity, plant interactions, and the potential use of biological inoculants to enhance plant growth and adaptation. Additionally, it is important for exploring the sources of biologically active molecules with industrial and medicinal significance. Fungal identification is challenging due to their vast diversity and morphological similarities among species. Therefore, molecular phylogeny methods are essential for accurate species identification. DNA barcoding systems utilize a short standard region (typically between 400 and 800 bp) for species identification, with the ITS region being a significant molecular marker known for its high accuracy in fungal identification. The ITS region has been widely used for a diverse range of fungi^14,15^. In this study, phylogenetic analysis revealed that the isolates were grouped into distinct clades, and their precise taxonomic placement was determined based on the ITS region analysis. The isolates obtained in this study belong to the Ascomycota branch, which is consistent with the dominance of Ascomycota species as endophytic fungi as shown in previous studies^16^. Among the studied samples, *Aspergillus* species (*A. bicephalus, A. fumigatus, A. niger, A. terreus*) and *Fusarium* species (*F. oxysporum, F. redolens*, and *F. solani*) exhibited the highest species richness. These species have been previously reported as endophytes in various hosts^17^. In this study, only *A. niger and T. allahabadensis* isolates formed a zone on a solid Sperber medium and were able to dissolve phosphate in a liquid Sperber medium. The phosphorus-dissolving ability of *A. niger* and *T. allahabadensis* has been previously documented^18,19^. Phosphate-dissolving fungi play a crucial role in breaking down insoluble phosphorus minerals in the soil by producing organic acids, thereby enhancing phosphorus availability to plants^20,21^. Wang et al. demonstrated that *A. niger* primarily dissolves phosphorus through the production of oxalic acid, tartaric acid, and citric acid^22^. The variations in the isolates’ phosphorus-dissolving capabilities, as indicated by the ratio of halo diameter to colony size, may be attributed to differences in the type, quantity, and release rate of organic acids produced by each isolate in the solid medium^23^. Additionally, some of these fungi can convert organic phosphorus in the soil into a mineral form that plants can absorb by producing phosphatase enzymes.

Phosphate-dissolving fungi enhance plant yield by increasing soil-soluble phosphorus. Despite constituting only 0.1 to 0.5% of the total fungal population in soil, phosphate-solubilizing fungi offer numerous advantages for plant nutrition. Fungi, with their hyphae-like structure, have a higher capacity to dissolve phosphate compared to bacteria, penetrating deeper into the soil^6^. Research indicates that P-solubilizing fungi release significantly higher concentrations of organic acids than bacteria, resulting in greater phosphorus-solubilizing activity. Additionally, endophytic fungi can dissolve all three common forms of phosphate (Ca-, Al-, and Fe-phosphate), making them valuable in both alkaline and acidic soils^24^. Consequently, they may serve as more effective phosphate solubilizers in soil than the rhizobacteria population.

The results of the present study revealed that all the studied fungal isolates produced indole acetic acid when grown in an L-tryptophan medium, as indicated by the pink color formation^25^. Specifically, endophytes capable of producing auxin exhibit a red/pink color change upon addition of Salkowski’s reagent, attributed to the interaction between auxin and iron resulting in complex compound formation. Previous studies have documented the auxin-producing abilities of *Aspergillus*^26^, *Talaromyces*^27^, *Fusarium*^28^, and *Alternaria*^29^ species. This study represents the first documentation of A. *bicephalus’s* capacity to produce auxin. Studies have shown that different fungal species have distinct pathways for indole-3-acetic acid (IAA) synthesis, with some species possessing multiple IAA synthesis pathways^30^. Previous research has demonstrated that IAA produced by endophytic fungi can stimulate the formation of lateral roots and promote the growth of hairy roots. Additionally, fungal-derived IAA can indirectly influence plants by boosting plant immune responses and suppressing pathogenic strains^31^. It has been reported that IAA produced by endophytic fungi can modulate gene expression and antioxidant homeostasis to mitigate disease. For instance, in a study on sesame plants, Cymbopogon et al. found that the endophytic fungus Penicillium sp. effectively alleviated oxidative stress induced by *Fusarium* sp. through IAA production^32^. In another investigation, pretreatment of tomatoes with IAA-producing Trichoderma significantly reduced wilt disease caused by *Ralstonia solanacearum*^33^.

Inoculating wheat seeds with endophytic fungi significantly increased the initial growth of wheat roots, likely due to the production of IAA by the endophytic strains. Previous studies have also reported the enhancement of crop growth by auxin-producing endophytic fungi^30^. However, in this study, the increase in wheat root growth did not show a strong correlation with the amount of auxin production by the strains. It is well-documented that microbial auxin secretion facilitates root colonization and the expression of other growth-promoting traits^34^. Furthermore, *A. fumigatus* endophytes have been reported to produce other growth regulators such as gibberellic acid and ACC deaminase enzyme^35^, which could explain the observed effect of inoculated endophytic fungi on wheat root growth.

## Conclusion

The high reactivity of phosphorus with soil and the formation of its insoluble forms significantly reduces the efficiency of using phosphorus chemical fertilizers in agricultural lands. Additionally, in drought conditions, reduced phosphorus mobility and suppressed root development severely limit plant access to phosphorus. In such circumstances, the isolates obtained in this study, particularly *A. niger*, can effectively enhance phosphorus availability and promote plant root system development through auxin hormone production. Further research on the fungal isolates for additional growth-promoting characteristics and their potential for colonizing crop roots is necessary to leverage this approach for sustainable production in the face of climate change.

## Materials and Methods

### Isolation of endophytes from T. ramosissima

Plant specimens of *T. ramosissima* were collected from natural habitats in Tabriz, Iran (N49.148204, E114.875597), in May 2021. Voucher specimen after identifying by Dr. Mostafa Ebadi (ASMUH-10331) was deposited at the herbarium of Azerbaijan Shahid Madani University. Root samples were washed with running tap water for 10 minutes to remove soil particles and cut into 1 cm sections. For surface sterilization, the root pieces were submerged in 70% ethanol for 1 minute, in a 0.5% sodium hypochlorite solution for 3 minutes, and in 70% ethanol for 30 seconds, then washed three times with sterile distilled water. They were allowed to dry on a paper towel. After drying, the root pieces were placed on potato dextrose agar (PDA) supplemented with 50 mg/L chloramphenicol to inhibit bacterial growth. All plates were incubated at 25°C for 7–10 days to isolate the endophytic fungi. Pure fungal isolates were obtained by selecting individual colonies from the PDA plates and transferring them to fresh PDA medium, then incubating at 25°C for 10 days.

### Molecular identification and phylogenetic analysis

For molecular identification, the pure isolates were inoculated into 100 ml of potato dextrose broth (PDB, Difco) media and incubated at 24°C for one week on a rotary shaker at 110 rpm. The mycelia were harvested by filtration, dried for 48 hours at 50°C, and stored at 4°C for further study. Total genomic DNA of the fungal isolates was extracted following the method proposed Zhu et al. ^36^ The purified genomic DNA was used as a template for rDNA amplification using primers ITS1 (5’-TCCGTAGGTGAACCTGCGG-3’) and ITS4 (5’-TCCTCCGCTTATTGATATGC-3’)^37^.

Amplification was carried out in a thermocycler (SensoQuest) with the following program: 94°C for 2 min, 35 cycles at 94°C for 20 s, 57°C for 30 s, 72°C for 2 min, followed by a final extension step of 10 min at 72°C. The PCR products were then electrophoresed at 85 V on 1.2% agarose gels, and the resulting bands were visualized under a UV transilluminator.

The obtained sequences were deposited in the NCBI GenBank and compared with existing sequences using BLAST searches. Phylogenetic analyses were conducted using MEGA software version 6.0, with sequence alignment performed using MUSCLE software^38^. The phylogenetic tree was constructed using the neighbor-joining (NJ) method with a p-distance substitution model and bootstrapping of 1000 replications.

### Qualitative and Quantitative Assessment of Phosphate Solubilization

The evaluation of fungi’s ability to solubilize phosphate was conducted on Sperber media, consisting of 10 g glucose, 0.5 g yeast extract, 0.1 g CaCl2, 0.25 g MgSO4.7H2O, 2.5 g Ca3(PO4)2, and 15 g agar dissolved in 1000 ml distilled water. Plugs of PDA with each fungal isolate were transferred to the centers of Sperber medium and incubated at 26°C, with observations made daily for 7 days. The growth of phosphate-solubilizing fungal colonies is indicated by the formation of a clear zone around the colony^39^.

The quantitative assessment of fungi’s phosphate solubilization ability was conducted using a colorimetric assay method^40^. A plug of the isolate was inoculated into 100 mL of liquid Sperber medium and incubated in a shaking incubator at 25°C and 120 rpm for 7 days. After centrifugation of the culture at 6000 rpm for 45 minutes to separate fungal mycelia from the supernatant, 1 ml of the supernatant was mixed with 1 ml Vanadate-molybdate reagent and incubated for 10 minutes at room temperature. The yellow color formation indicated phosphate concentration, which was measured using a spectrophotometer at a wavelength of 470 nm.

### Quantitative Estimation of Indole-3-Acetic Acid Production

The measurement of IAA was conducted using a colorimetric method described by Gordon and Weber^41^. Czapek Dox (CD) medium supplemented with 5 mg/l L-tryptophan was used for this purpose. In 100 mL Erlenmeyer flasks containing 20 mL CD medium, each flask was inoculated with 5 mm fungal plugs and incubated at room temperature for 6 days on a rotary shaker at 120 rpm. After centrifugation of the flask contents at 6000 rpm for 10 minutes, 1 mL of the supernatant of each isolate was mixed with 2 mL of Salkowski reagent (HClO4 (70%) + FeCl_3_ (0.5M)) and incubated in the dark for 30 minutes. The development of a pink color indicated IAA production, with absorbance measured at 530 nm using a spectrophotometer. The IAA concentration was determined using an IAA standard curve generated from serial dilutions of IAA solution.

### Impact of Endophytic Fungal Inoculation on Root Growth

The endophytic fungal isolates (T3-T17) were used as bio-inoculants to evaluate their effect on wheat plant (*Triticum aestivum* L.) root growth, measured as root length (cm). Wheat seeds were surface-sterilized with 2.5% sodium hypochlorite, washed with distilled water, soaked in CD broth media (supplemented with 5 mg/l L-tryptophan) inoculated with fungal isolates, and incubated for 24 hours. The soaked seeds were then transferred to sterilized cups with wet filter paper and incubated for 7 days at room temperature in the dark. CD broth without fungal inoculations served as the control. The average root length of wheat was calculated for each treatment based on ten samples.

### Statistical Analysis

Each experiment was replicated three times, and data were analyzed using SPSS ver. 17 with one-way analysis of variance (ANOVA) in a completely randomized design. Mean comparisons were determined using Tukey’s test (p < 0.05).

## Figures and Tables

**Table 1:** Morphological characters of endophytic fungi isolated from T. ramosissima on PDA.

Morphotype	Colony character				
	Shape	Surface texture	Growth rate (cm)	Obverse color	Reverse color
T3	Circular	Glabrous	2.2 ± 0.1	White green	Yellow orange
T4	Circular	Cottony	3.1 ± 0.2	Black	Sunny green
T5	Irregular	Floccose	2.3 ± 0.2	White pink	Pink
T7	Irregular	Glabrous	2.1 ± 0.1	White	Orange
T8	Irregular	Granular	6.1 ± 0.4	Silver grey	Black
T11	Circular	Cottony	7.3 ± 0.2	White	Canary
T12	Irregular	Floccose	4.8 ± 0.2	Plum	Yellow orange
T15	Circular	Granular	3.7 ± 0.1	Bright green	Cream
T17	Circular	Cottony	5.2 ± 0.2	White	Tan

**Table 2:** The ITS sequence identification of the endophytic fungal isolates from T. ramosissima

Fungal Isolate Code	GenBank Accession Number	Homolog Sequences	Sequence Identity %	Closest Accession Number
T3	OR345323	*Talaromyces allahabadensis*	100	KP281439.1
T4	OR345324	*Aspergillus niger*	100	MT152319.1
T5	OR345325	*Fusarium oxysporum*	100	MG564296.1
T7	OR345326	*Aspergillus bicephalus*	99.79	LT601380.1
T8	OR345327	*Alternaria alternata*	100	OR018293.1
T11	OR345328	*Fusarium solani*	100	MT638068.1
T12	OR345329	*Aspergillus terreus*	100	MT558939.1
T15	OR345330	*Aspergillus fumigatus*	99.82	MT635279.1
T17	OR345331	*Fusarium redolens*	100	MT516323.1
